# Health status and health behaviors among citizen endurance Nordic skiers in the United States

**DOI:** 10.1186/s13104-017-2619-4

**Published:** 2017-07-24

**Authors:** Paul J. Anderson, Ralph S. Bovard, Mohammad Hassan Murad, Timothy J. Beebe, Zhen Wang

**Affiliations:** 1 Mayo Clinic Division of Preventive, Occupational, and Aerospace Medicine, 200 1st Street SW, Rochester, MN 55905 USA; 2 Health Partners Occupational and Environmental Medicine Residency, 205 S. Wabasha Street, St. Paul, MN 55107 USA; 3 Robert D. and Patricia E. Kern Center for the Science of Health Care Delivery, Mayo Clinic, 200 1st Street SW, Rochester, MN 55905 USA; 4 Mayo Clinic Division of Health Care Policy and Research, Mayo Clinic Division of Health Sciences Research, Rochester, MN USA; 5 HealthPartners Department of Occupational and Environmental Medicine, 5100 Gamble Dr Ste 100, Saint Louis Park, MN 55416 USA

**Keywords:** Obesity, Overweight, Physical activity, Nordic skiing, Cross-country skiing, Health behaviors, Health status

## Abstract

**Background:**

More than two-thirds of Americans are overweight or obese (69%) placing them at high risk for a wide array of chronic diseases. Physical activity anchors most approaches to obesity prevention and weight management, but physical activity levels remain low in the general US population. As a group, citizen athletes who compete in Nordic skiing events such as the American Birkebeiner participate in fitness cultures that promote physical activity.

**Methods:**

During October–November 2014, we emailed a 48 question online survey to 23,611 individuals who had participated in the American Birkebeiner ski event, the largest citizen ski race in North America. Descriptive statistics were used to summarize data. Binomial and student t test were used to compare binary and continuous outcomes to health behaviors of the US population.

**Results:**

5433 individuals responded. Obesity prevalence (BMI ≥30) was 3% and average BMI was 24. Skiers reported very good health (88%), higher fitness than peers (99%), freedom from depression (93%) low levels of smoking (3%), high consumption of fruits and vegetables, moderate alcohol use, and high levels of physical activity. Fifteen percent practiced all 4 healthy living characteristics known to reduce cardiovascular event risk.

**Conclusions:**

As a group, citizen endurance Nordic skiers enjoy low levels of obesity, below average BMI, and report lifestyle behaviors known to decrease obesity, promote health, and reduce cardiovascular disease risk. Future research should explore hypotheses that explain how the fitness cultures surrounding citizen athletic events support weight loss, cardiovascular fitness, and healthy lifestyle habits.

## Background

According to the Centers for Disease Control and Prevention, more than two-thirds (69%) of Americans are overweight (BMI = 25–29) and more than one-third (35.6%) of adults are obese (BMI ≥30) placing them at high risk for some of the leading causes of preventable death including cardiovascular disease, stroke, type 2 diabetes, and some cancers [1]. In 2008 currency, annual obesity cost the United States was $147 billion dollars and on average obese persons generated $1429 more in annual health care costs than normal weight individuals [2].

Physical activity interventions play a central role in obesity prevention and reduction efforts along with dietary choices [3]. Regular physical activity reduces body fat and has also been shown to reduce all-cause premature death [4, 5]. Nevertheless, fewer than half of all US adults (48%) report that they meet 2008 physical activity guidelines of 150 min of exercise per week and even fewer (10%) meet these guidelines when they are objectively measured with an accelerometer [6]. 25% of Americans report no leisure time physical activity, whatsoever [7].

Citizen athletes who participate in the fitness cultures surrounding endurance sporting events represent a subgroup of the US population who reverse these trends. Each year in the United States, millions of citizen athletes participate in formal endurance competitions such as running races, cycling races, triathlons, swimming races, cross-country and alpine ski races, and distance walking events. It is known that runners display significant reduction in cardiovascular mortality from following recommendations for healthy lifestyle behaviors, but to our knowledge the heightened health status and health behavior patterns have not been widely documented in other sports, including Nordic skiing [8].

In this cross-sectional study, we surveyed self-reported BMI, health status, and health behaviors among a group of citizen endurance athletes (Nordic skiers participating in an American Birkebeiner race). Through these observations, we wanted to generate hypotheses that would explore the elevated health status of individuals who are linked through their participation in a distinctive fitness culture created by a popular endurance race such as the American Birkebeiner. We also compared this group to objective and self-reported health behaviors the US general population as a point of reference.

The American Birkebeiner ski race (aka “The Birkie”) was founded in 1973 and it is now the largest Nordic ski race in North America attracting over 12,000 participants in 2015. The American Birkebeiner Ski Foundation champions the race as a “year round lifestyle choice for outdoor fitness enthusiasts of all levels”. In addition to Nordic ski races, Birkie events now include a fall mountain-bike marathon, spring and summer running races on the Birkebeiner trail, family centered ski weekends, skijoring events (skiing while pulled by a dog) and a fat-tire on-snow bicycle race. The hallmark Birkebeiner events are the 54 km classic ski race and the 52 km skate ski race, as well as the 25 km Korteloppet which may be completed in skate or classic technique.

## Methods

The study was approved by the Mayo Clinic Institutional Review Board prior to implementation. Between October 30, 2014 and December 8, 2014, 23,611. Individuals who had ever participated in at least one ski event sponsored by the American Birkebeiner Ski Foundation received an email invitation to participate in an online survey and gave written consent to use their results by agreeing to fill out the survey. Each participant in a Birkie event receives a unique “Birkie ID number” which was coupled with their email address to form an individual participant record.

### Data collection

The online survey explored demographics, health status, and health behaviors. Health status and health behavior questions were modeled after the Behavioral Risk Factor Surveillance System (BRFSS) and questions commonly used in the National Health and Nutrition Examination Survey (NHANES). Health status questions explored how participants would rate their current health status, how they felt their health compared with others, and whether they had any chronic conditions relevant to sport participation. Health behavior questions explored topics such as nutrition, exercise habits, sleep patterns, smoking history, and alcohol use. Self-reported demographic and physiologic indicators were collected in order to calculate BMI.

### Statistical analysis

We conducted descriptive analyses to summarize the survey respondents’ demographics, biometrics, health status, and health behaviors. We then compared the survey results to the results from US population using NHANES 2011–2012 and the 2014 Mortality and Morbidity Weekly Report (MMWR) Surveillance summary of data from BRFSS [9]. Binomial probability test was used for proportion outcomes and student t test for continuous outcomes. Tests were deemed statistically significant if two tailed *P* < 0.05. All statistical analyses were conducted using STATA ver. 13.1 (StataCorp, College Park, TX).

## Results

Survey responses were received by 5433 participants (response rate = 23%) and were included in analysis. Relative to the US population, Birkie skiers tended to be older (mean age = 47.9 vs. 37.2) male (64.5% vs. 48.85) married (71.36% vs. 53.1%) report that they had a college education (86.2% vs. 31%) and maintained full time employment (66.42% vs. 29.4%). More than half of all Birkie respondents reported a household income >$100,000 dollars (54.2% vs. 23.7%). The mean BMI for Birkie skiers was 23.8, lower than the US population which has a mean BMI = 26.7. Very few Birkie skiers (3.23%) had a BMI >30 compared with 27.7% of the NHANES US population. Please see Table 1 for details.

**Table 1:** Demographics and biometrics for respondents to the Birkie Health Survey, 2015

	Birkebeiner skiers (SD)	US population (SE)^a^	P value
Mean age	47.9 (0.21)	37.2 (0.30)	<0.001
% Male gender	64.5	48.8	<0.001
Mean BMI	23.8	26.7	<0.001
% BMI ≥30	3.23	27.7	<0.001
% Married	71.36	53.1	<0.001
% Employed full time (>35 h)	66.42	29.4	<0.001
% 4 year college education or more	86.2	31.0	<0.001
% Household income >$100,000	54.2	23.7	<0.001

^a^National Health and Nutrition Examination Study (NHANES)

When asked about their health status, 99.1% of Birkie participants reported that they were as fit or more fit than their peers and 88.3% reported very good or excellent health status. Compared with the US population, a higher percentage of Birkie respondents reported freedom from physical limitations (82.8% vs. 76.4%) and freedom from depression or low mood (93.1% vs. 82.2%).

Questions about health behaviors revealed that only 18.2% of Birkie participants reported eating less than 1 fruit daily, and only 14.4% reported eating vegetables less than once daily. According to the CDC State Indicator Report on fruit and vegetable consumption, 39.2% of the US population reports eating fruit less than once daily and 23.1% report eating vegetables less than once daily [10]. Birkie participants also reported lower levels of current smoking than the US population (3.37% vs. 21.2%) and lower levels of binge drinking (10.35% vs. 18.3%). 25% of the US population reports no leisure time physical activity, but this number was 5.84% among Birkie respondents. This information along with other health behaviors is displayed in Table 2.

**Table 2:** Self-reported health status and health behaviors: 2015 American Birkebeiner Survey Participants vs. US population

	Birkebeiner skiers (%)	US population	P value
Health status			
Very good/excellent health status	88.3	82.8% [9]	<0.001
As fit/more fit than peers	99.1	–	
No physical limitations	82.8	76.4% [9]	<0.001
Free from depression/low mood	93.1	82.2% [9]	<0.001
Health behaviors			
% Sleep <7 h/night	65.1	35.3% [11]	0.55
% Snoring	36.3	48.0% [11]	<0.001
% Unintentionally falling asleep	31.3	37.9% [11]	<0.001
% Eating fruit less than 1 time daily	18.2	39.2% [10]	<0.001
% Eating vegetables less than 1 time daily	14.4	23.1% [10]	<0.001
Current smokers	3.37	21.2% [9]	<0.001
Drink alcohol	90.5	87.6% [9]	<0.001
Moderate alcohol consumption (0–2 drinks per occasion)	83.9	–	
Binge drinking, ever	10.3	18.3% [9]	<0.001
No leisure time physical activity	5.84	25.5%	<0.001
Arthritis	18.7	24.4% [9]	<0.001

## Discussion

Relative to the general US population, American Birkebeiner Survey participants display very low levels of obesity, lower than average BMI, high self-reported health status, and a high prevalence of healthy lifestyle behaviors, including regular physical activity.

The prevalence of obesity in the United States is currently estimated at 35.6%, and with a national mean BMI of 26.7, the average American is overweight (BMI between 25 and 29.9) [1]. In contrast, the average BMI for Birkie participants is 23.8 and the number of Birkebeiner participants with a BMI ≥30 is only 3.23%. While the low incidence of obesity among Birkie skiers may be due in large part to self-selection by current athletes, we hypothesize that participation in a race culture may play help individuals who are trying initiate and maintain weight loss.

**Fig. 1:**
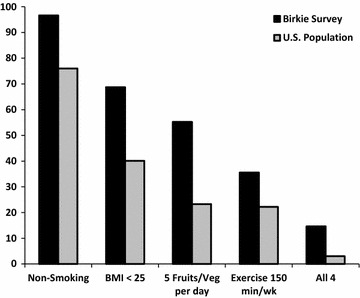
Prevalence of four healthy lifestyle characteristics (HLCs): Birkie Survey vs. US population [12, 13]

The health benefits of physical activity may to extend to other healthy behaviors as well. Several studies have shown that individuals can reduce their risk of heart attack or stroke by between 40 and 80% if they adopt four healthy lifestyle characteristics (HLCs): (1) don’t smoke, (2) eat five fruits and vegetables per day, (3) maintain a BMI <25, and (4) exercise >150 min/week. Yet, only about 3% of the US population adheres to all four HLCs [12, 13]. Not only do our data show that Birkie participants report higher prevalence of each of the HLCs than the general population, but nearly five times as many (15%) report routine adherence to all four of these healthy lifestyle behaviors which suggests a potential reduction in the risk of cardiovascular events among Birkie athletes (see Fig. 1). We hypothesize that the incidence of cardiovascular events among Birkebeiner skiers would be lower than non-skiers in the US population.

Our data underscore the commonly observed coexistence of vigorous physical activity and higher levels of other beneficial health behaviors [14–16]. Skiers in the American Birkebeiner participate in a communal fitness lifestyle that may discourage behaviors such as smoking, excess alcohol use, poor diet, overweight and obesity, and physical inactivity, and that may encourage physical activity and a host of other healthy behaviors which improve the ability to perform well in community fitness events. We hypothesize that participation in the fitness culture surrounding the American Birkebeiner reinforces healthy lifestyle behaviors relative to non-participation.

### Strengths and limitations

This study is the first to report health behaviors of Nordic skiers in the United States and one of the few that describe these health habits in endurance athletes. We had a large sample size that allowed evaluation of multiple factors. We modeled our survey to measure constructs similar to those in national surveys to allow comparison and hypothesis generation.

Nordic skiing can be costly and our study participants reported higher socioeconomic status, higher levels of educational attainment, and more leisure time for training. The Nordic origins of cross-country skiing and the trails in the United States where it can be practiced currently attract predominantly white participants although there are exceptions to this trend. While Birkie skiers appear to have fewer barriers to physical activity related to cost, sport knowledge, ease of access, and leisure time, these demographic realities do not negate the high levels of physical activity and health behaviors reported in this subgroup of the US population. Healthy individuals are more likely to participate in Nordic ski races which introduces a source of bias that tempers the conclusion that race cultures alone strictly cause improved fitness.

Application of these results to individuals with different demographics and background must be made with caution. Disadvantaged populations may not experience the low levels of obesity and high prevalence of healthy lifestyle behaviors reported here either because of pre-existing conditions, because participation in endurance sports is not the cultural norm, or because the barriers to preparation and participation for endurance events are currently insurmountable. Also, we were unable to collect physiological data and we did not solicit information on formal medical diagnoses besides mood disorders and arthritis. This limits our ability to objectively describe physical activity, adverse health outcomes among citizen endurance athletes and leaves us to speculate based on risk reduction estimates reported in the literature regarding self-reported health behaviors.

These survey results are important for preventive medicine and public health because they document elevated self-reported health outcomes in a group defined by participation in a fitness culture. These individuals report elevated adherence to preventive health recommendations for physical activity, diet, and other healthy lifestyle characteristics. Another result of the survey is the identification of powerful fitness cultures that often surround competitive endurance events such as the American Birkebeiner. Finally, the influence that fitness cultures may exert on individuals and communities adds weight to preventive medicine and public health guidance that emphasizes group activities over individualized plans for health behavior change.

## Conclusions

This study displays a high level of self-reported healthy behaviors among participants in the physical activity culture generated by the American Birkebeiner. Citizen endurance athletes may offer a beacon of hope amidst the rising tide of health conditions related to sedentary lifestyles and obesity in the United States and internationally. We suggest that the apparent fitness cultures created by events like the American Birkebeiner may be potent motivators for behavior change in the realm of physical activity that may otherwise be unavailable. Organizations that host, promote, supply, and financially sponsor such events may be serving as valuable public health change agents. Future research into citizen endurance athlete cultures should focus on understanding the attitudes, social-support patterns, training techniques, and health cultures created by large citizen athletic events and explore hypotheses that explain how fitness cultures promote weight loss, cardiovascular health, and health lifestyle habits.

## Abbreviations

BMI: body mass index
BRFSS: Behavioral Risk Factor Surveillance System
HLC: healthy lifestyle characteristics
MMWR: Mortality and Morbidity Weekly Report
NHANES: National Health and Nutrition Examination Study
